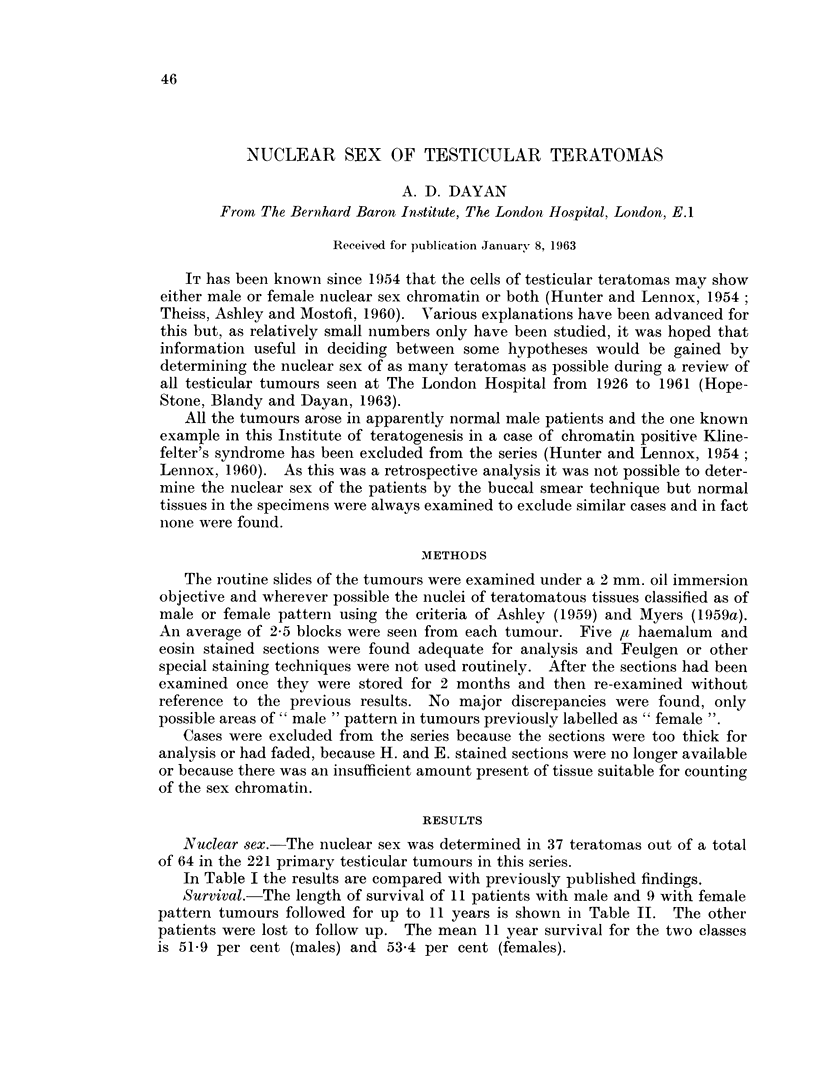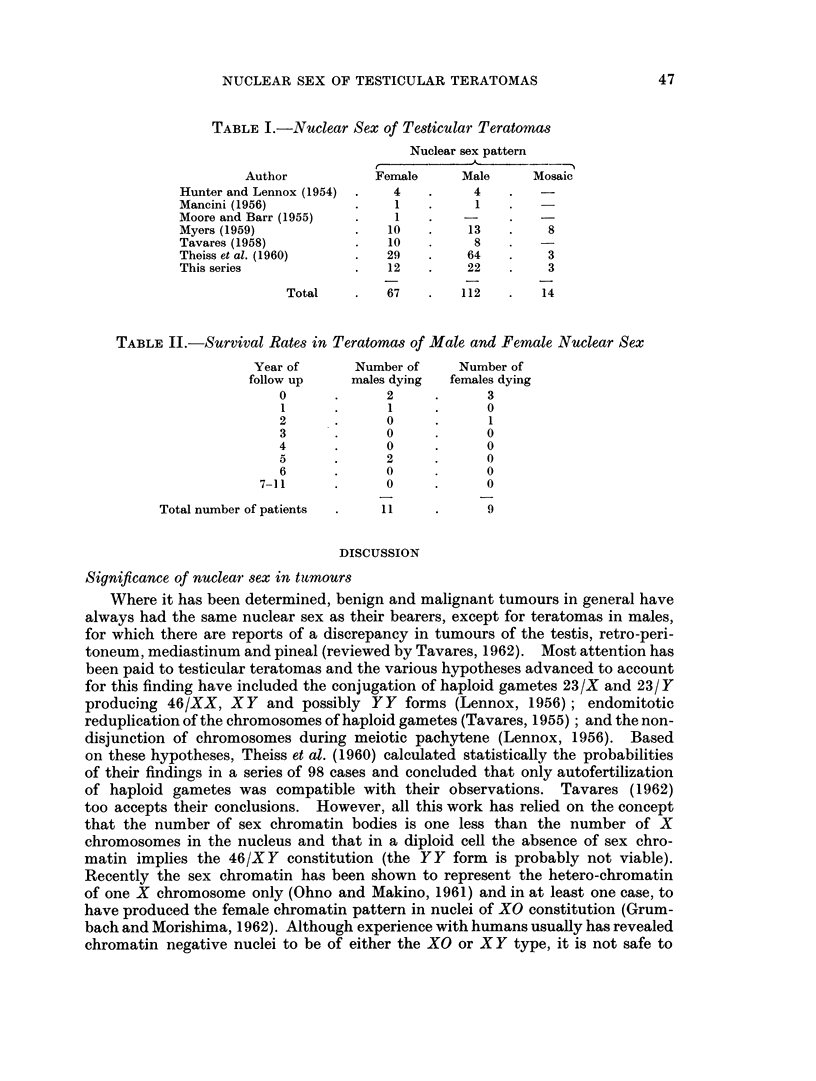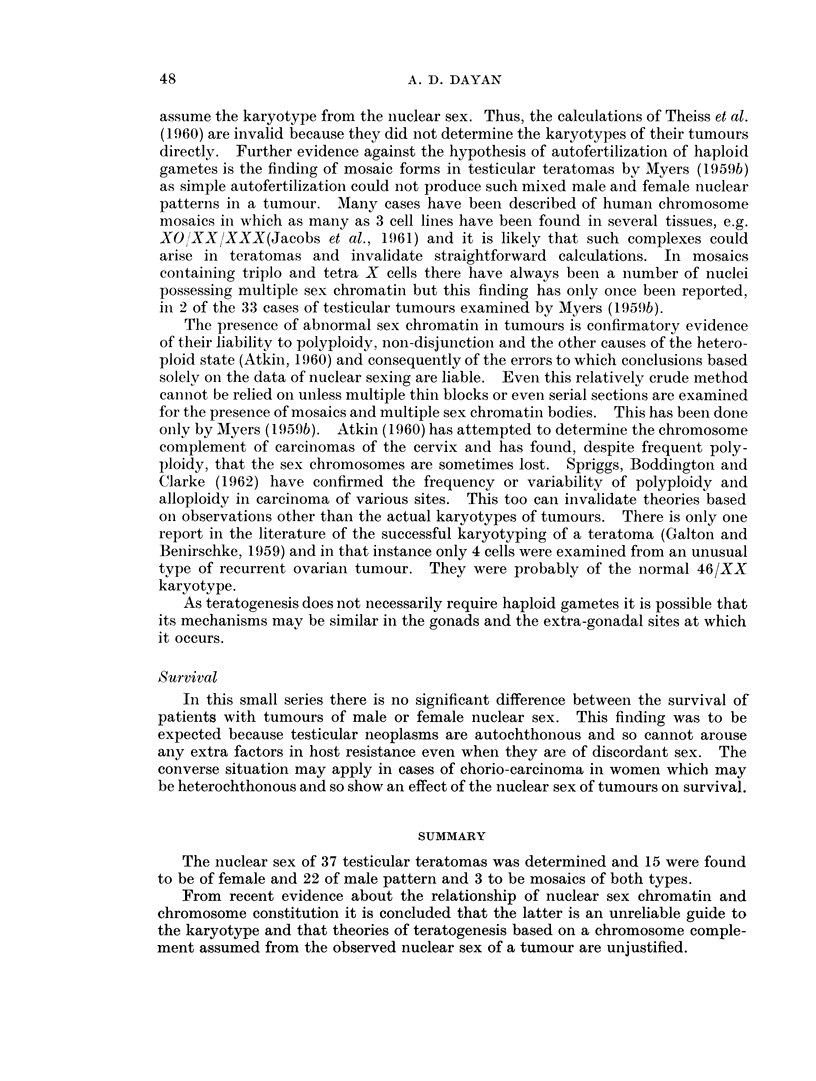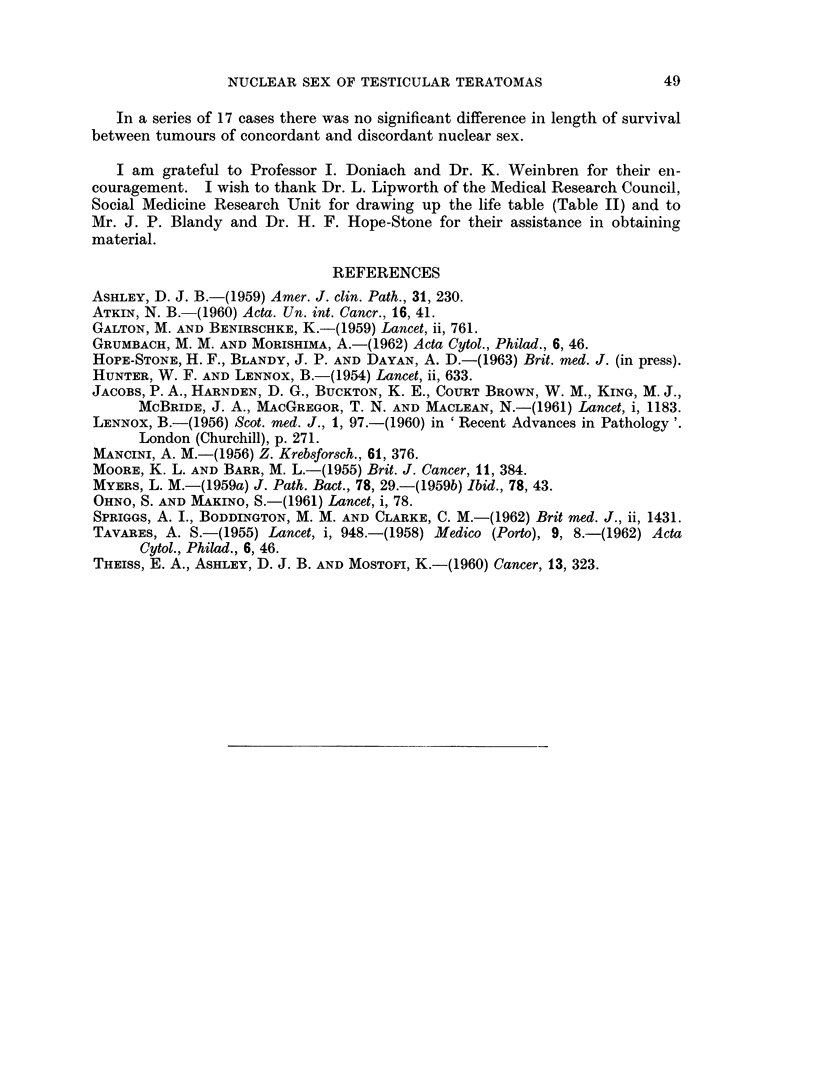# Nuclear Sex of Testicular Teratomas

**DOI:** 10.1038/bjc.1963.6

**Published:** 1963-03

**Authors:** A. D. Dayan


### NUCLEAR SEX OF TESTICULAR TERATOMAS

A. D. DAYAN

*From The Bernhard Baron Institute, The London Hospital, London, E.*1

Received for publication January 8, 1963

It has been known since 1954 that the cells of testicular teratomas may show either male or female nuclear sex chromatin or both (Hunter and Lennox, 1954; Theiss, Ashley and Mostofi, 1960). Various explanations have been advanced for this but, as relatively small numbers only have been studied, it was hoped that information useful in deciding between some hypotheses would be gained by determining the nuclear sex of as many teratomas as possible during a review of all testicular tumours seen at The London Hospital from 1926 to 1961 (Hope-Stone, Blandy and Dayan, 1963).

All the tumours arose in apparently normal male patients and the one known example in this Institute of teratogenesis in a case of chromatin positive Klinefelter's syndrome has been excluded from the series (Hunter and Lennox, 1954; Lennox, 1960). As this was a retrospective analysis it was not possible to determine the nuclear sex of the patients by the buccal smear technique but normal tissues in the specimens were always examined to exclude similar cases and in fact none were found.

### METHODS

The routine slides of the tumours were examined under a 2 mm. oil immersion objective and wherever possible the nuclei of teratomatous tissues classified as of male or female pattern using the criteria of Ashley (1959) and Myers (1959*a*). An average of 2·5 blocks were seen from each tumour. Five $\mu$ haemalum and eosin stained sections were found adequate for analysis and Feulgen or other special staining techniques were not used routinely. After the sections had been examined once they were stored for 2 months and then re-examined without reference to the previous results. No major discrepancies were found, only possible areas of " male " pattern in tumours previously labelled as " female ".

Cases were excluded from the series because the sections were too thick for analysis or had faded, because H. and E. stained sections were no longer available or because there was an insufficient amount present of tissue suitable for counting of the sex chromatin.

### RESULTS

*Nuclear sex.*—The nuclear sex was determined in 37 teratomas out of a total of 64 in the 221 primary testicular tumours in this series.

In Table I the results are compared with previously published findings.

*Survival.*—The length of survival of 11 patients with male and 9 with female pattern tumours followed for up to 11 years is shown in Table II. The other patients were lost to follow up. The mean 11 year survival for the two classes is 51·9 per cent (males) and 53·4 per cent (females).

TABLE I.—*Nuclear Sex of Testicular Teratomas*

| Author | Nuclear sex pattern: Female | Male | Mosaic |
|---|---|---|---|
| Hunter and Lennox (1954) | 4 | 4 | — |
| Mancini (1956) | 1 | 1 | — |
| Moore and Barr (1955) | 1 | — | — |
| Myers (1959) | 10 | 13 | 8 |
| Tavares (1958) | 10 | 8 | — |
| Theiss *et al.* (1960) | 29 | 64 | 3 |
| This series | 12 | 22 | 3 |
| Total | 67 | 112 | 14 |

TABLE II.—*Survival Rates in Teratomas of Male and Female Nuclear Sex*

| Year of follow up | Number of males dying | Number of females dying |
|---|---|---|
| 0 | 2 | 3 |
| 1 | 1 | 0 |
| 2 | 0 | 1 |
| 3 | 0 | 0 |
| 4 | 0 | 0 |
| 5 | 2 | 0 |
| 6 | 0 | 0 |
| 7–11 | 0 | 0 |
| Total number of patients | 11 | 9 |

### DISCUSSION

*Significance of nuclear sex in tumours*

Where it has been determined, benign and malignant tumours in general have always had the same nuclear sex as their bearers, except for teratomas in males, for which there are reports of a discrepancy in tumours of the testis, retro-peritoneum, mediastinum and pineal (reviewed by Tavares, 1962). Most attention has been paid to testicular teratomas and the various hypotheses advanced to account for this finding have included the conjugation of haploid gametes $23/X$ and $23/Y$ producing $46/XX$, $XY$ and possibly $YY$ forms (Lennox, 1956); endomitotic reduplication of the chromosomes of haploid gametes (Tavares, 1955); and the non-disjunction of chromosomes during meiotic pachytene (Lennox, 1956). Based on these hypotheses, Theiss *et al.* (1960) calculated statistically the probabilities of their findings in a series of 98 cases and concluded that only autofertilization of haploid gametes was compatible with their observations. Tavares (1962) too accepts their conclusions. However, all this work has relied on the concept that the number of sex chromatin bodies is one less than the number of $X$ chromosomes in the nucleus and that in a diploid cell the absence of sex chromatin implies the $46/XY$ constitution (the $YY$ form is probably not viable). Recently the sex chromatin has been shown to represent the hetero-chromatin of one $X$ chromosome only (Ohno and Makino, 1961) and in at least one case, to have produced the female chromatin pattern in nuclei of $XO$ constitution (Grumbach and Morishima, 1962). Although experience with humans usually has revealed chromatin negative nuclei to be of either the $XO$ or $XY$ type, it is not safe to assume the karyotype from the nuclear sex. Thus, the calculations of Theiss *et al.* (1960) are invalid because they did not determine the karyotypes of their tumours directly. Further evidence against the hypothesis of autofertilization of haploid gametes is the finding of mosaic forms in testicular teratomas by Myers (1959*b*) as simple autofertilization could not produce such mixed male and female nuclear patterns in a tumour. Many cases have been described of human chromosome mosaics in which as many as 3 cell lines have been found in several tissues, e.g. $XO/XX/XXX$(Jacobs *et al.*, 1961) and it is likely that such complexes could arise in teratomas and invalidate straightforward calculations. In mosaics containing triplo and tetra $X$ cells there have always been a number of nuclei possessing multiple sex chromatin but this finding has only once been reported, in 2 of the 33 cases of testicular tumours examined by Myers (1959*b*).

The presence of abnormal sex chromatin in tumours is confirmatory evidence of their liability to polyploidy, non-disjunction and the other causes of the heteroploid state (Atkin, 1960) and consequently of the errors to which conclusions based solely on the data of nuclear sexing are liable. Even this relatively crude method cannot be relied on unless multiple thin blocks or even serial sections are examined for the presence of mosaics and multiple sex chromatin bodies. This has been done only by Myers (1959*b*). Atkin (1960) has attempted to determine the chromosome complement of carcinomas of the cervix and has found, despite frequent polyploidy, that the sex chromosomes are sometimes lost. Spriggs, Boddington and Clarke (1962) have confirmed the frequency or variability of polyploidy and alloploidy in carcinoma of various sites. This too can invalidate theories based on observations other than the actual karyotypes of tumours. There is only one report in the literature of the successful karyotyping of a teratoma (Galton and Benirschke, 1959) and in that instance only 4 cells were examined from an unusual type of recurrent ovarian tumour. They were probably of the normal $46/XX$ karyotype.

As teratogenesis does not necessarily require haploid gametes it is possible that its mechanisms may be similar in the gonads and the extra-gonadal sites at which it occurs.

### *Survival*

In this small series there is no significant difference between the survival of patients with tumours of male or female nuclear sex. This finding was to be expected because testicular neoplasms are autochthonous and so cannot arouse any extra factors in host resistance even when they are of discordant sex. The converse situation may apply in cases of chorio-carcinoma in women which may be heterochthonous and so show an effect of the nuclear sex of tumours on survival.

### SUMMARY

The nuclear sex of 37 testicular teratomas was determined and 15 were found to be of female and 22 of male pattern and 3 to be mosaics of both types.

From recent evidence about the relationship of nuclear sex chromatin and chromosome constitution it is concluded that the latter is an unreliable guide to the karyotype and that theories of teratogenesis based on a chromosome complement assumed from the observed nuclear sex of a tumour are unjustified.

In a series of 17 cases there was no significant difference in length of survival between tumours of concordant and discordant nuclear sex.

I am grateful to Professor I. Doniach and Dr. K. Weinbren for their encouragement. I wish to thank Dr. L. Lipworth of the Medical Research Council, Social Medicine Research Unit for drawing up the life table (Table II) and to Mr. J. P. Blandy and Dr. H. F. Hope-Stone for their assistance in obtaining material.

### REFERENCES

ASHLEY, D. J. B.—(1959) *Amer. J. clin. Path.*, **31**, 230.
ATKIN, N. B.—(1960) *Acta. Un. int. Cancr.*, **16**, 41.
GALTON, M. AND BENIRSCHKE, K.—(1959) *Lancet*, ii, 761.
GRUMBACH, M. M. AND MORISHIMA, A.—(1962) *Acta Cytol., Philad.*, **6**, 46.
HOPE-STONE, H. F., BLANDY, J. P. AND DAYAN, A. D.—(1963) *Brit. med. J.* (in press).
HUNTER, W. F. AND LENNOX, B.—(1954) *Lancet*, ii, 633.
JACOBS, P. A., HARNDEN, D. G., BUCKTON, K. E., COURT BROWN, W. M., KING, M. J., McBRIDE, J. A., MACGREGOR, T. N. AND MACLEAN, N.—(1961) *Lancet*, i, 1183.
LENNOX, B.—(1956) *Scot. med. J.*, **1**, 97.—(1960) in ' Recent Advances in Pathology '. London (Churchill), p. 271.
MANCINI, A. M.—(1956) *Z. Krebsforsch.*, **61**, 376.
MOORE, K. L. AND BARR, M. L.—(1955) *Brit. J. Cancer*, **11**, 384.
MYERS, L. M.—(1959*a*) *J. Path. Bact.*, **78**, 29.—(1959*b*) *Ibid.*, **78**, 43.
OHNO, S. AND MAKINO, S.—(1961) *Lancet*, i, 78.
SPRIGGS, A. I., BODDINGTON, M. M. AND CLARKE, C. M.—(1962) *Brit med. J.*, ii, 1431.
TAVARES, A. S.—(1955) *Lancet*, i, 948.—(1958) *Medico* (*Porto*), **9**, 8.—(1962) *Acta Cytol., Philad.*, **6**, 46.
THEISS, E. A., ASHLEY, D. J. B. AND MOSTOFI, K.—(1960) *Cancer*, **13**, 323.